# *In vitro* Study of *Lactobacillus paracasei* CNCM I-1518 in Healthy and *Clostridioides difficile* Colonized Elderly Gut Microbiota

**DOI:** 10.3389/fnut.2019.00184

**Published:** 2019-12-10

**Authors:** Sophie Fehlbaum, Christophe Chassard, Clarissa Schwab, Maarja Voolaid, Candice Fourmestraux, Muriel Derrien, Christophe Lacroix

**Affiliations:** ^1^Laboratory of Food Biotechnology, Institute of Food, Nutrition and Health, ETH Zurich, Zurich, Switzerland; ^2^Danone Nutricia Research, Palaiseau, France

**Keywords:** *Lactobacillus paracasei* CNCM I-1518, *Clostridioides difficile*, gut microbiota, intestinal model, metataxonomics, metatranscriptomics, *Faecalibacterium*, elderly

## Abstract

Consumption of probiotic bacteria can result in a transient colonization of the human gut and thereby in potential interactions with the commensal microbiota. In this study, we used novel PolyFermS continuous fermentation models to investigate interactions of the candidate probiotic strain *Lactobacillus paracasei* CNCM I-1518 (*L. paracasei*) with colonic microbiota from healthy elderly subjects using 16S rRNA gene amplicon sequencing and metatranscriptomics, or with microbiota *in vitro*-colonized with *Clostridioides difficile* (*C. difficile* NCTC 13307 and *C. difficile* DSM 1296)—an enteropathogen prevalent in the elderly population. Small changes in microbiota composition were detected upon daily addition of *L. paracasei*, including increased abundances of closely related genera *Lactobacillus* and *Enterococcus*, and of the butyrate producer *Faecalibacterium*. Microbiota gene expression was also modulated by *L. paracasei* with distinct response of the *Faecalibacterium* transcriptome and an increase in carbohydrate utilization. However, no inhibitory effect of *L. paracasei* was observed on *C. difficile* colonization in the intestinal models under the tested conditions. Our data suggest that, in the *in vitro* experimental conditions tested and independent of the host, *L. paracasei* has modulatory effects on both the composition and function of elderly gut microbiota without affecting *C. difficile* growth and toxin production.

## Introduction

The large intestine is the most densely populated site of the human body with over 10^14^ microbial cells. This diverse microbial community exerts functions that are important to maintain host health, including energy, and nutrients supply by fermentation of otherwise indigestible food components, development of a balanced immune system and the protection against pathogens, termed colonization resistance (1, 2). Different diseases have been associated with compositional changes in intestinal communities (2) and a disruption of the healthy microbial communities (also referred to dysbiosis) can result in the loss of colonization resistance and an overgrowth of pathogens, such as *Clostridium difficile* (3), recently renamed *Clostridioides difficile* (4).

Apart from disease, diet and medications are important modulators of the gut microbiota. However, the gut microbiota also changes throughout lifespan and it is suggested that the aging-associated differences in gut microbiota might be linked to the general decline in the health status (5–8). Old age was associated with a decrease in potentially beneficial bacteria, including bifidobacteria (9), and *Faecalibacterium prausnitzii*, and an increase in facultative anaerobes such as enterobacteria (10–12). Furthermore, the risk of *C. difficile* infection (CDI) is elevated in old age following antibiotic treatment (13). Decreases in short chain fatty acids (SCFA) production have also been described for the elderly (9, 14).

Probiotics are defined as “live micro-organisms which, when administered in adequate amounts, confer a health benefit on the host” (15). Specific probiotic strains have been shown to promote colonization resistance and are promising adjunct therapy for the treatment of gastrointestinal infections, such as CDI (16–18). A meta-analysis based on 6,261 subjects reported that incidence of *C. difficile* was lower for subjects who consumed probiotic than of controls. Notably, a better efficacy was observed when probiotics were administered closer to the first antibiotic dose (19).

Mechanisms of probiotic action include the direct interaction with the commensal gut microbiota, inhibition of enteric pathogens or their metabolites, and modulation of the immune system (20). Probiotics have been associated with improved clinical outcome in several studies (21) but the effect of probiotics on gut microbiota composition and especially on the functional activity is not always known. *Lactobacillus* strains are often used as probiotics due to their technological properties and the general assumption that they are safe as they have been traditionally used in fermented dairy products (22). *Lactobacillus paracasei* CNCM I-1518 (*L. paracasei*) is a candidate probiotic strain that belongs to the *Lactobacillus casei* group (consisting of *L. casei, L. paracasei* subspecies *paracasei*, and *L. rhamnosus*). This strain can survive gastrointestinal transit and to modulate immune function (23–27). Fermented milk product containing *L. paracasei* CNCM I-1518 was associated with a decreased duration of common gastrointestinal and respiratory infections (28), and consumption of fermented milk containing the same strain reduced the incidence of antibiotic- and *C. difficile*-associated diarrhea in elderly patients taking antibiotics (29). Recently, this *L. paracasei* strain was administered to intensive care unit patients for prevention of antibiotic associated diarrhea and CDI. The trial was of a small sample size and it was found that one patient in the probiotic group developed CDI compared to three in the control group (30).

Assessing the effect of probiotics on the gut microbiota composition and activity can be difficult due to the hindered accessibility of the gastrointestinal tract. *In vitro* models simulating the human colon represent a useful tool for mechanistic studies on the interactions of probiotics with the gut microbiota and pathogens independent of the host (31, 32).

We recently developed *in vitro* fermentation models on the novel PolyFermS platform (33) with elderly immobilized fecal microbiota for investigations of *C. difficile* colonization and antibiotic treatment testing (34, 35). In this study, we assessed the potential of *L. paracasei* to modulate the composition and function of elderly gut microbiota reproduced in these *in vitro* colonic continuous fermentation models of the PolyFermS platform with and without *C. difficile* inoculation on the composition and activity of microbial communities and their functional properties using 16S rRNA gene amplicon sequencing and metatranscriptomics, respectively. To investigate associations observed in modeled microbiota, we also performed single and co-cultures of *L. paracasei, F. prausnitzii*, and *C. difficile*.

## Methods

### Bacterial Strains

*L. paracasei* CNCM I-1518 was provided by Danone Research (Palaiseau, France). *Faecalibacterium prausnitzii* DSM 17677 was purchased from the Deutsche Sammlung von Mikroorganismen und Zellkulturen (DSMZ, Braunschweig, Germany). *C. difficile* DSM 1296 (PCR ribotype 001) and *C. difficile* NCTC 13307 (PCR ribotype 012) were purchased from DSMZ and the National Collection of Type Cultures (NCTC, Salisbury, United Kingdom), respectively.

For inoculation of colonic fermentation studies *L. paracasei* and vegetative cells of *C. difficile* DSM 1296 were cultured from glycerol stocks (33%, −80°C) at 37°C in serum flasks flushed with N_2_ and CO_2_ at 3:1 ratio or using the anaerobic Hungate culturing technique (36) containing fermentation medium simulating human chyme as previously described (34). Spores of *C. difficile* DSM 1296 and NCTC 13307 were prepared according to Sorg and Dineen (37) as previously described (35).

Yeast extract-casein hydrolysate-fatty acids (YCFA) medium (38) was used to routinely culture the bacterial strains in anaerobic Hungate tubes at 37°C for co-culture studies of *L. paracasei* with either *F. prausnitzii* or *C. difficile* DSM 1296. YCFA was supplemented with glucose, soluble starch and cellobiose (Sigma-Aldrich Chemie GmbH, Buchs, Switzerland), each at a concentration of 2 g L^−1^ (YCFA-GSC).

### Continuous Colonic Fermentation Setup

**Figure 1:**
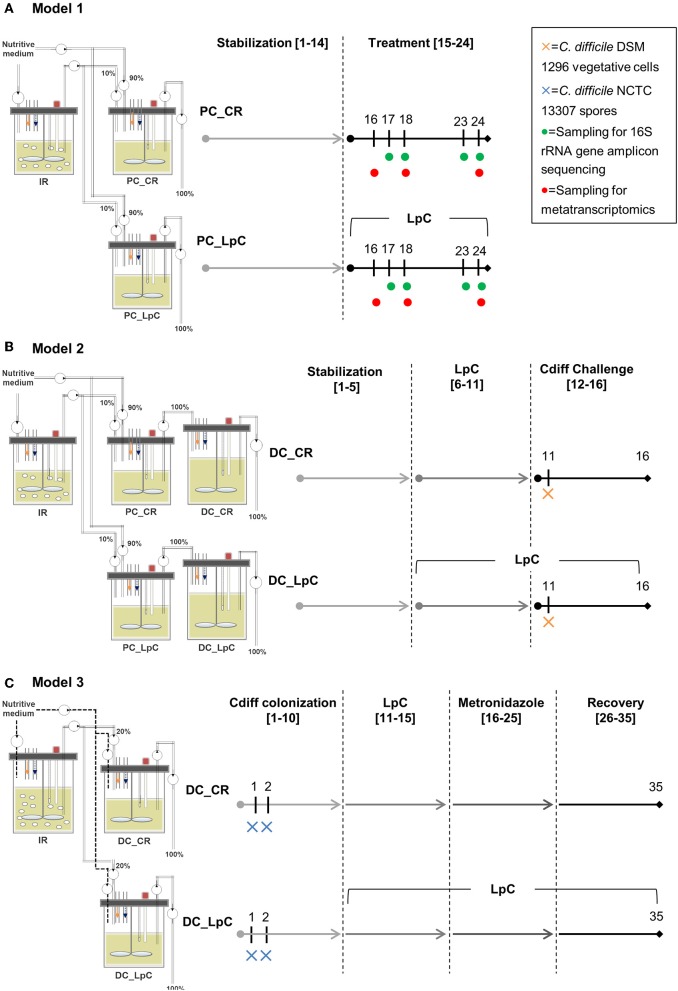
Experimental timeline of continuous colonic fermentation models. **(A)** Model 1. *L. paracasei* (LpC)-treated reactor mimicking the proximal colon section (PC_LpC) was inoculated with *L. paracasei* twice daily at days 15–24. Sampling for 16S rRNA gene amplicon sequencing was performed on days 17, 18, 23, and 24. Sampling for metatranscriptomics analysis was done on days 16, 18, and 24. **(B)** Model 2. *L. paracasei* (LpC)-treated reactor mimicking the proximal colon section (PC_LpC) was inoculated with *L. paracasei* twice daily at days 6–16; *C. difficile* DSM 1296 vegetative cells were inoculated on day 11 into DC_CR and DC_LpC mimicking the distal colon section. **(C)** Model 3. *C. difficile* NCTC 13307 spores were inoculated on day 1 and 2 into DC_CR and DC_LpC mimicking the distal colon section; DC_LpC was treated with *L. paracasei* twice daily at days 11–35; DC_CR and DC_LpC were treated with metronidazole (days 16–25) and recovery was observed at days 26–35. In all models sampling for qPCR and HPLC was performed daily. IR, inoculum reactor; PC, proximal colon; DC, distal colon; CR, control reactor; LpC, *L. paracasei* CNCM I-1518.

All three continuous *in vitro* fermentation models investigated in this study were based on the PolyFermS design and are displayed in Figure 1. The PolyFermS model allows the parallel testing of different treatments on singular microbiota as described previously (33). Common to all three models was an inoculum reactor (IR, 37°C, retention time of 9 h, pH 5.7) inoculated with single donor fecal microbiota immobilized in gellan-xanthan beads. Model 1 and 3 were inoculated with fecal microbiota obtained from the same elderly donor (75–80 years old) with a 7-months interval. Model 2 was inoculated with fecal microbiota from a different donor (70–75 years old) (34, 35). Fecal donors did not receive antibiotic treatment for at least 3 months prior to sample collection and did not consume probiotics for at least 1 month before fecal sampling. An informed written consent was obtained from both donors. A fermentation medium simulating human chyme was used in all three models, as presented previously (35). Different models were used for the experiments as previously described in details (34, 35). In model 1 run at conditions mimicking the proximal colon (PC, 37°C, retention time 9 h, pH 5.7), the IR was connected in parallel to a control reactor (PC_CR) and test reactor treated with *L. paracasei* (PC_LpC). PC_CR and PC_LpC were continuously inoculated with 10% effluent from IR and 90% fresh fermentation medium. The IR of model 2 was connected in parallel to two sets of two-stage reactors, mimicking the proximal (PC, 37°C, retention time of 9 h, pH 5.7) and transverse-distal colon (DC, 37°C, retention time of 18 h, pH 6.8). The two sets consisted of a control (PC_CR + DC_CR) and *L. paracasei* test reactors (PC_LpC + DC_LpC). The PC reactors were continuously fed with 10% effluent from IR and 90% fresh fermentation medium while DC reactors received 100% effluent from the respective PC reactor. The IR of the model 3 was used to feed 100% one control (DC_CR) and one *L. paracasei* test reactor (DC_LpC) operated at transverse-distal colon conditions (37°C, retention time of 25 h, pH 6.8). During antibiotic treatment and recovery period in model 3, control and test reactors were fed with fresh fermentation medium to avoid the inflow of untreated microbiota.

### Experimental Design of Colonic Models

In model 1, the effect of *L. paracasei* on the healthy elderly proximal colonic microbiota was investigated. A stabilization period of 14 days was performed before PC_LpC was inoculated with *L. paracasei* twice daily for 10 days (Figure 1). *L. paracasei* was prepared from an overnight culture which was centrifuged (6,000 g, 5 min). The pellet was re-suspended in fresh fermentation medium and inoculated with a syringe to obtain final concentrations of around log_10_ 7.5 cells mL^−1^ that corresponds to the approximate number of living *L. paracasei* cells detected in stool samples following ingestion (24). Microbiota composition was analyzed with 16S rRNA gene amplicon sequencing on four selected days at the beginning and at the end of *L. paracasei* treatment (days 16, 17, 23, and 24, Figure 1). qPCR was performed during the last days of stabilization period and throughout the treatment period of selected bacterial groups that were impacted by *L. paracasei* according to 16S rRNA amplicon sequencing or metatranscriptomics. The metatranscriptome was analyzed on 3 days corresponding to the beginning, middle, and end of *L. paracasei* treatment (days 16, 18, and 24). The metabolic activity was assessed with high performance liquid chromatography with refractive index detection (HPLC-RI) during the three last days of stabilization period and throughout the *L. paracasei* treatment period.

Model 2 was previously described for development of elderly microbiota models and for *C. difficile* colonization investigations (34, 35). After an initial stabilization phase of 18 days (34) and treatment periods for *C. difficile* investigations (35), control and test reactors were exchanged with new reactors, that were connected to IR for stabilization phase of 5 days before treatment with *L. paracasei* was started (Figure 1). *L. paracasei* was inoculated twice daily into the test system 2 (PC_LpC + DC_LpC) for 11 days (days 6–16). On day 11, DC_CR and DC_LpC were inoculated once with vegetative cells of *C. difficile* DSM 1296. The *C. difficile* cells were prepared from an overnight culture which was centrifuged (6,000 g, 5 min). The pellet was re-suspended in fresh fermentation medium and inoculated with a syringe to obtain final concentrations of approximately log_10_ 6 cells mL^−1^. Reactor effluents of the DC reactors were collected 6 h post *C. difficile* inoculation and afterwards daily to determine cell numbers of *L. paracasei* and *C. difficile* as well as cytotoxin titers.

In model 3, the effects of *L. paracasei* on *C. difficile* NCTC 13307 were investigated in transverse-distal colon conditions before, during and after metronidazole treatment (Figure 1). The *C. difficile* NCTC 13307 strain was chosen due to a better colonization of reactors upon spore inoculation as described before (35). Both DC reactors were instilled with *C. difficile* spores at a concentration of 10^7^ cfu, which were added once on two consecutive days at the beginning of *C. difficile* colonization period. *L. paracasei* treatment was performed in reactor DC_LpC from day 10 of fermentation with twice daily addition of *L. paracasei* cells throughout the remaining days of fermentation as described above for model 1. Metronidazole (Sigma-Aldrich) treatment was performed twice daily at a final concentration of 333 mg L^−1^ at days 16–25 in DC_CR and DC_LpC. Reactor effluents of DC's were collected daily for qPCR analysis of *L. paracasei* and *C. difficile* abundance, and for cytotoxin determination using Vero cell analysis.

The effect of ceftriaxone and metronidazole on *C. difficile* spore germination and colonization, respectively, as well as the general effect of these antibiotics on the gut microbiota was presented earlier (35).

### Co-cultures of *L. paracasei* and *F. prausnitzii* DSM 17677

Growth of *F. prausnitzii* was investigated in co-culture with *L. paracasei* because an increase in relative abundance of the genus *Faecalibacterium* was observed during *L. paracasei* treatment in PC_LpC reactor of model 1 by 16S rRNA gene amplicon sequencing. Culturing was performed in Hungate tubes containing 10 mL YCFA-GSC medium. For each measurement point, individual tubes were inoculated with 2% of three overnight cultures. Optical density (OD_600nm_), pH and metabolites were analyzed at 0, 8, 48, and 72 h of incubation. qPCR analysis was performed to determine cell numbers of *L. paracasei* and *F. prausnitzii* as described below. Metabolite concentrations were assessed from culture supernatants using HPLC-RI analysis. Strains were also grown individually for comparison. The co-culture test was performed four times with three replicates each time and average values of the four tests are presented.

### Co-cultures of *L. paracasei* and C. *difficile* DSM 1296

*L. paracasei* and *C. difficile* DSM 1296 were investigated in co-cultures to assess the effect of *L. paracasei* on *C. difficile* growth and toxin production in the absence of complex microbiota. Strains were grown in Hungate tubes containing 10 mL YCFA-GSC medium and for each intended measurement time point separate tubes were inoculated with 2 or 4%, of *C. difficile* and *L. paracasei* overnight cultures, respectively. Because *L. paracasei* grew slower than *C. difficile, L. paracasei* was inoculated first and *C. difficile* was added after 5 h. OD, pH and metabolites were determined at 0, 5, 10, 13, and 25 h. Cell counts were additionally determined by plating on Wilkins-Chalgren agar (Oxoid AG) supplemented with cysteine-HCL and resazurin (Sigma-Aldrich). Serial dilutions were prepared in an anaerobic chamber and plates were incubated at 37°C in anaerobic jars (BioMérieux Suisse SA). Colonies of *L. paracasei* and *C. difficile* were distinguished by different colony morphology. *L. paracasei* had milky appearance, smooth surface, colony diameter (2–5 mm) larger than for *C. difficile*, round with entire margin. In contrast, *C. difficile* exhibited yellow ground-glass appearance, ruffled edges and smaller colonies. Toxin production was assessed after 13 and 25 h incubation in co-cultures and compared to single cultures using the Vero cell assay test. The co-culture test was performed twice with three replicates each time, and average values of the two tests are presented.

### DNA Extraction and qPCR Analysis

Genomic DNA was extracted from 2 mL fermentation effluent and co-cultures using the FastDNA SPIN Kit for Soil (MP Biomedicals, Illkirch, France). Abundance of specific bacterial and archaeal groups or species were measured in duplicate on an ABI PRISM 7500-PCR (Applied Biosystems, Zug, Switzerland) using a reaction volume of 25 μl as described before (39). All assays were carried out using the 2 × SYBR Green PCR Master Mix (Applied Biosystems). Specific primers were used for enumeration of different bacterial groups, including *L. paracasei, F. prausnitzii*, and *C. difficile* (Table S1). A factor of 6 and 10 was used^1^ to calculate the number of cells for *F. prausnitzii* and *C. difficile*, respectively, to account for several copies of 16S rRNA gene (40). Standard curves preparation and reaction conditions were described previously (41).

### 16S rRNA Gene Amplicon Sequencing

16S rRNA gene amplicon sequencing of effluent samples of colonic model 1 was carried out at DNAVision (Gosselies, Belgium) on a 454 Life Sciences Genome Sequencer FLX instrument (Roche Applied Science, Vilvoorde, Belgium). Amplification of the V5-V6 hypervariable 16S rRNA region was performed using primers 784F and 1061R (42). Data was analyzed using the open source software package Quantitative Insights Into Microbial Ecology (QIIME), v1.9 (43) as described before (35).

### RNA Isolation and Metatranscriptome Sequencing

One mL effluent samples of colonic model 1 was collected directly from reactors and mixed with 1 mL 60% glycerol at −40°C, kept on ice for 20 min and centrifuged for 15 min (3,220 × g, 4°C). The supernatant was discarded, and the pellet was shock-frozen in liquid nitrogen and stored at −80°C. For total RNA isolation, pellets were re-suspended in 400 μL cold Man, Rogosa and Sharpe medium (MRS) supplemented with cysteine at 0.5 g/l and transferred to a screw cap tube containing 500 μL chlorophorm/phenol (1:1, v/v), 30 μL SDS 10% (44), 30 μL 3 M Na-acetate and 400 mg zirconium beads (0.1 mm). The mixture was disrupted in a bead beater (4 × 40 s, 5 m s^−1^) with cooling on ice between cycles and centrifuged for 12 min (12,000 × g, 4°C). The supernatant was added to 200 μL ice cold chloroform, centrifuged again as before and from the resulting supernatants RNA was isolated using the High Pure RNA Isolation Kit (Roche Diagnostics, Switzerland) according to the manufacturer's instructions. RNA concentrations and quality were determined on a NanoDrop 1000 spectrophotometer (Thermo Fisher Scientific, Washington, USA) and on an Agilent 2100 Bioanalyzer (Agilent, Basel, Switzerland), respectively. Paired-end RNA-seq using an Illumina HiSeq 2500 v4 was conducted at the Functional Genomics Center Zurich (ETH Zurich, Switzerland). RNA libraries were prepared using TruSeq RNA stranded library preparation kit and standard protocols supplied by Illumina.

For bioinformatics analysis, a pipeline consisting of SortMeRNA (45) for separation of rRNA and mRNA, and FLASH (46) for overlapping the paired-end sequences were used. rRNA sequences (100,000) were compared to the modified SILVA database provided by CREST (47). Putative mRNA reads were compared to the NCBI RefSeq database using MALT (http://ab.inf.uni-tuebingen.de/software/malt/) which is based on DIAMOND (48). Transcripts were taxonomically classified using MEGAN (49). Putative mRNA reads were also uploaded to MG-RAST for functional classification according to the SEED Subsystem scheme using default settings.

### HPLC-RI Analysis

Acetate, butyrate, propionate, formate, and lactate were determined in fermentation effluent and co-culture samples by HPLC in duplicate (Thermo Fisher Scientific Inc. Accela, Wohlen, Switzerland). Sample supernatants were filtered into vials through a 0.45 μm nylon HPLC filter (Infochroma AG, Zug, Switzerland). The analysis was run at a flow rate of 0.4 mL min^−1^ using an Aminex HPX-87H (Bio-Rad Laboratories AG, Reinach, Switzerland) or Rezex ROA-Organic Acid column (Phenomenex, Basel, Switzerland), for effluent and co-culture samples, respectively and 10 mM H_2_SO_4_ as eluent. A refractive index detector was used for detection.

### Vero Cell Analysis

*C. difficile* cytotoxin production was monitored in effluent samples of colonic models 2 and 3 and in samples of co-culture test of *L. paracasei* with *C. difficile* DSM 1296 using a Vero cell cytotoxicity assay as described before (35).

### Statistical Analysis

Statistical analyses of co-culture studies were done using JMP 10.0 (SAS Institute, USA). All data are expressed as mean ± SD of several co-culture tests performed in triplicate in batch fermentation studies. Growth (log_10_-transformed), pH values, metabolites, and toxin production were compared between pure and co-cultures using the non-parametric Kruskal-Wallis test. Statistical analyses of metatranscriptomics data were done using a one-tailed Student's *t*-test for relative abundance comparisons between control reactor and *L. paracasei* reactor.

### Data Availability

All 454-pyrosequencing files have been deposited to the National Center for Biotechnology Information (NCBI) Sequence Read Archive (SRA) under bioproject accession number SRP144222. The mRNA reads are available at MG-RAST under project ID: IFT_antibiotics.

## Results

### Effect of *L. paracasei* on the Gut Microbiota Structure in Proximal Colon Conditions

In a first model mimicking proximal colon (model 1), we investigated the impact of 10-days inoculation of *L. paracasei* on the gut microbiota composition of a healthy elderly donor using 16S rRNA gene sequencing. In the control reactor (PC_CR), Firmicutes and Bacteroidetes were the dominant phyla, while Proteobacteria and Actinobacteria represented <6% of the community. Clostridiales were the dominant bacterial order with *Lachnospiraceae* and *Ruminococcaceae* contributing the majority of reads (Figure S1), *Lactobacillaceae* represented between 0.9 and 2.7% of effluent microbiota; a mean relative abundance of 2.4% of *Lactobacillus* spp. relative to total bacterial 16S rRNA genes was determined using qPCR.

**Figure 2:**
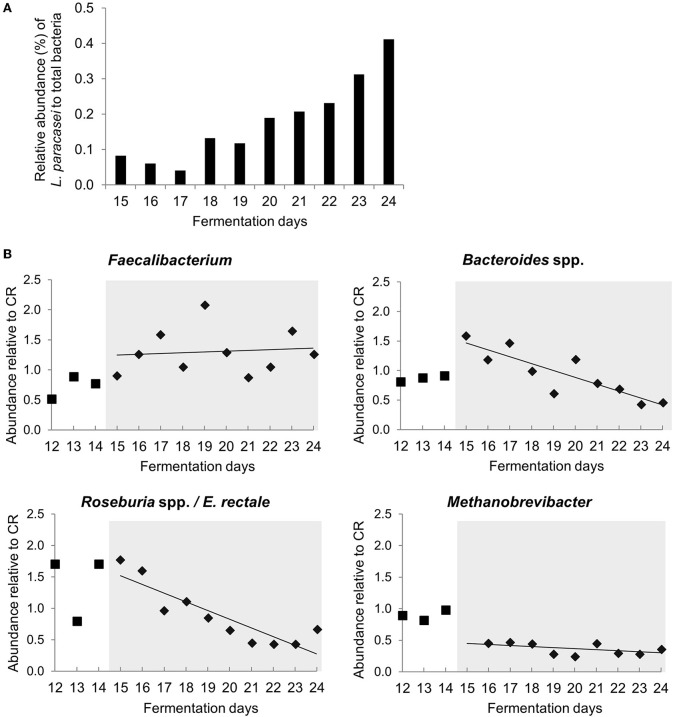
Abundance of selected bacterial groups in proximal colon section of model 1 assessed with qPCR **(A)** *L. paracasei* abundance compared to total bacteria in PC_LpC during treatment period. **(B)** Relative abundance of *Faecalibacterium, Bacteroides* spp., *Roseburia* spp. */Eubacterium rectal*e and *Methanobrevibacter* in PC_LpC (16S rRNA genes target taxon relative to 16S rRNA genes total bacteria) normalized to relative abundance in PC_CR during last 3 days of stabilization period (days 12–14,■) and during treatment period (days 15–24,♦).

**Figure 3:**
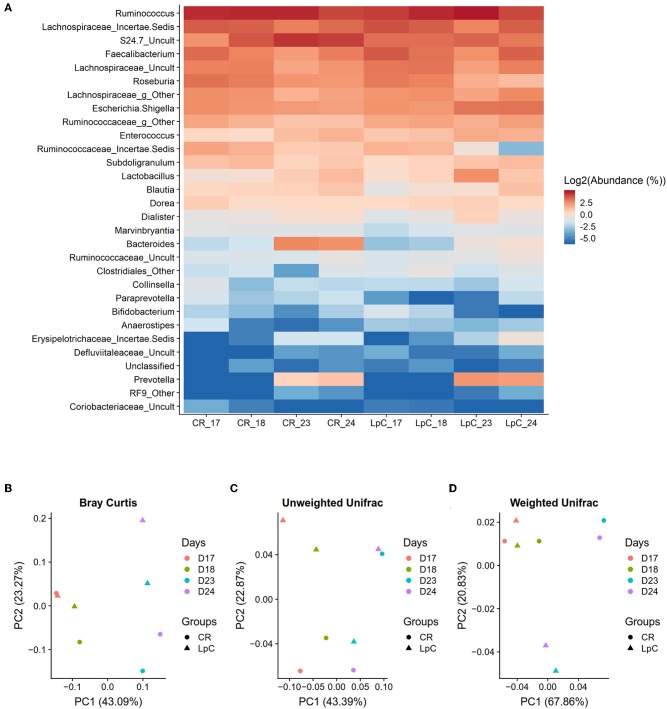
Effect of *L. paracasei* supplementation on microbiome composition in (LpC)-treated reactor mimicking the proximal colon section (PC_LpC) of model 1 assessed with 16S rRNA gene amplicon sequencing on days 17, 18, 23, and 24. **(A)** Heatmap showing the relative abundance at genus level of PC_LpC compared to PC_CR. **(B)** PCoA analysis of Bray-Curtis distances in PC_LpC and PC_CR. **(C)** PCoA analysis of unweighted UniFrac distances in PC_LpC and PC_CR. **(D)** weighted UniFrac distances in PC_LpC and PC_CR.

*L. paracasei* was not detected during stabilization phase in PC_CR and PC_LpC, and in PC_CR during treatment using strain specific qPCR (Table S1). However, in the inoculated reactor, addition of *L. paracasei* at log_10_ 8 cfu mL^−1^ led to progressive increase of relative abundance of the strain, from 0.1 up to 0.4% after 10 days (Figure 2A). The addition of *L. paracasei* in proximal colon had little impact on the relative abundance of major phyla, however, analysis of days 17, 18, 23, and 24 at the beginning and end of the treatment showed an increase of *Lactobacillus, Faecalibacterium, Ruminococcaceae, and Enterococcus*, in PC_LpC compared to control reactor (PC_CR) (Figure 3A). At the same time, the addition of *L. paracasei* was associated with decreased abundance of *Roseburia, Ruminococcaceae incertae sedis, Bacteroides*, and *Paraprevotella*. The increase of *Faecalibacterium* and decrease in *Bacteroides* spp. and *Roseburia* spp./*E. rectale* after the addition of *L. paracasei* was confirmed by qPCR (Figure 2B).

Beta-diversity was assessed by the Bray-curtis distances, unweighted UniFrac distances, and weighted UniFrac distances (Figures 3B–D). A clear separation of the reactor with *L. paracasei* at days 23 and 24 and all the other samples was observed with weighted UniFrac (Figure 3D).

### Effect of *L. paracasei* on Gene Expression and Metabolite Formation in Proximal Colon Conditions

Next, we evaluated the impact of *L. paracasei* inoculation on activity of the gut microbiota in the same experience using metatranscriptome analysis. RNA sequencing of fermentation effluents yielded between 4.5 and 12.8 million overlapped reads (average size 153 ± 31 bp), between 7.4 and 10.7% of those reads were identified as putative mRNA transcripts (225,000 and 1.57 million reads) (Table S2).

#### Microbiota Composition of the Metatranscriptome

**Figure 4:**
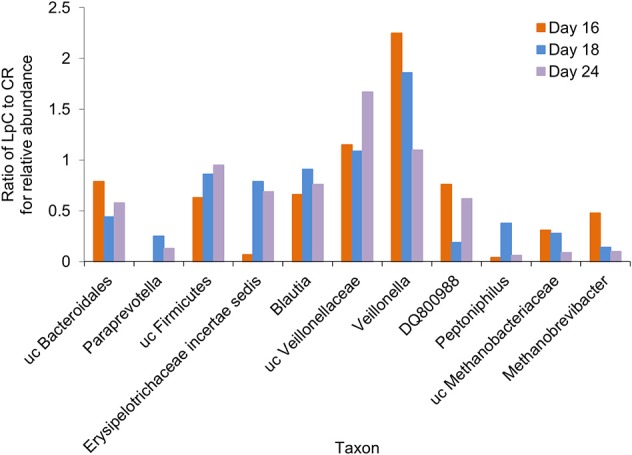
Ratio of the relative abundance of taxonomic groups between the *L. paracasei* (LpC)-treated reactor and the control reactor (CR) mimicking the proximal colon section (model 1) assessed with metatranscriptomics on days 16, 18, and 24. Only taxonomic groups that differed in abundance between PC_CR and PC_LpC are shown.

A subset of 100,000 rRNA reads per samples was used for taxonomic classification. Bacteria and archaea were detected in the effluents of the *in vitro* fermentation model (Figure 4 and Table S3). Clostridiales were the dominant bacterial order (78–89% of rRNA reads and 66–73% of mRNA reads, Tables S3, S4), with *Ruminococcaceae* (38–49%), and *Lachnospiraceae* (23–27%) contributing the majority of transcripts (Tables S3, S4) and confirming results obtained by 16S rRNA gene amplicon sequencing (Figure S1). Relative abundance of unclassified *Veillonellaceae* and *Veillonella* were increased in the presence of *L. paracasei* while *Paraprevotella* and *Peptoniphilus* decreased (Figure 4). Methanobacteriales contributed between 0.01 and 0.04% of 16S rRNA transcripts. Relative abundance of unclassified *Methanobrevibacteriaceae* and *Methanobrevibacter* also decreased in reactors to which *L. paracasei* had been added. This decrease after *L. paracasei* addition could be confirmed by qPCR (Figure 2B). Taxonomic classification of mRNA reads was consistent with the community structure revealed by rRNA analysis (Table S3).

#### Functional Activity of the Microbiota

Between 74,166 and 1.7 million reads were assigned to SEED categories using MG-RAST. Almost 40% of all transcripts belonged to SEED categories “Carbohydrate metabolism” and “Protein metabolism.”

The addition of *L. paracasei* had modest impact on the relative abundance of most SEED categories (Table 1). However, relative abundance of transcripts assigned to SEED category “Membrane transport” was significantly (*p* < 0.05) increased when *L. paracasei* was present in the fermentation vessel due to the enhanced transcription of fructose and mannose, galactose, and sucrose specific sugar phosphotransferase systems [(PTS) Tables 1, 2]. As these categories were all related to sugar transport, we further investigated contributors to the SEED category “Carbohydrate Metabolism.” Subcategories “Di- and Oligosaccharides,” and “Fermentation” were consistently slightly enhanced on all 3 days tested when *L. paracasei* was added compared to controls. Within “Di- and Oligosaccharides,” sucrose phosphorylase (EC2.4.1.7), sucrose-6-phosphate hydrolase (EC3.2.1.26) and “Alpha-galactosidase” were 1.2–5 fold more abundant compared to controls. Within category “Fermentation,” “Butanol biosynthesis” (Pyruvate formate-lyase (EC 2.3.1.54), 1.2–1.8 fold) was the only noted change.

**Table 1:** Relative abundance of SEED categories Level 1 in control and *L. paracasei* (LpC)-treated reactor mimicking proximal colon section (model 1).

**Day**	**16**			**18**			**24**		
**Relative abundance (%)**	**CR**	**LpC**	**Ratio LpC/CR**	**CR**	**LpC**	**Ratio LpC/CR**	**CR**	**LpC**	**Ratio LpC/CR**
Amino acids and derivatives	8.22	8.13	0.99	8.32	8.28	1.00	8.49	8.53	1.01
Carbohydrates	19.48	19.22	0.99	19.57	20.36	1.04	20.78	20.86	1.00
Cell division and cell cycle	1.03	0.97	0.94	1.01	0.89	0.88	0.89	0.91	1.02
Cell wall and capsule	2.09	2.16	1.03	1.93	1.91	0.99	2.08	1.84	0.88
Clustering-based subsystems	13.66	14.17	1.04	14.13	14.00	0.99	14.05	13.71	0.98
Cofactors, Vitamins, Prosthetic Groups, Pigments	4.97	5.20	1.05	4.63	4.76	1.03	4.88	4.77	0.98
DNA metabolism	2.11	2.16	1.03	2.14	1.90	0.89	1.90	1.98	1.04
Dormancy and sporulation	0.16	0.13	0.81	0.15	0.17	1.13	0.14	0.12	0.81
Fatty Acids, Lipids, and Isoprenoids	2.99	3.11	1.04	2.59	2.65	1.02	2.55	2.48	0.97
Iron acquisition and metabolism	0.34	0.28	0.80	0.34	0.27	0.79	0.37	0.37	1.02
**Membrane Transport**	**2.30**	**2.56**	**1.11**	**2.21**	**2.38**	**1.08**	**2.35**	**2.71**	**1.15**
Metabolism of aromatic compounds	1.17	1.45	1.24	1.26	1.26	1.00	1.34	1.35	1.01
Miscellaneous	5.36	5.27	0.98	5.53	5.62	1.02	5.28	5.20	0.98
Motility and chemotaxis	0.58	0.86	1.47	0.61	0.62	1.01	0.62	0.53	0.86
Nitrogen metabolism	0.43	0.24	0.55	0.23	0.22	0.99	0.26	0.21	0.83
Nucleosides and nucleotides	2.64	2.48	0.94	2.73	2.73	1.00	2.80	2.55	0.91
Phages, Prophages, Transposable elements, Plasmids	1.13	1.08	0.95	1.05	0.97	0.92	1.13	1.14	1.01
Phosphorus metabolism	0.13	0.12	0.98	0.11	0.11	0.94	0.13	0.13	0.99
Photosynthesis	0.01	0.02	1.35	0.01	0.01	0.95	0.01	0.01	1.13
Potassium metabolism	0.10	0.08	0.85	0.11	0.08	0.76	0.10	0.11	1.13
Protein metabolism	19.88	19.27	0.97	20.32	20.17	0.99	19.05	19.51	1.02
RNA metabolism	3.81	3.57	0.94	3.75	3.52	0.94	3.42	3.40	0.99
Regulation and cell signaling	0.82	0.86	1.05	0.75	0.76	1.01	0.80	0.80	0.99
Respiration	2.21	2.12	0.96	2.16	2.16	1.00	2.28	2.30	1.01
Secondary metabolism	0.61	0.75	1.23	0.76	0.68	0.90	0.75	0.74	0.99
Stress response	2.26	2.18	0.97	2.15	2.04	0.95	1.92	2.18	1.13
Sulfur metabolism	0.35	0.34	0.97	0.33	0.35	1.05	0.40	0.37	0.93
Virulence, Disease and Defense	1.15	1.22	1.06	1.12	1.12	1.00	1.22	1.19	0.97

*In bold: SEED categories that are increased in PC_LpC relative to PC_CR (p < 0.05)*.

**Table 2:** Relative abundance of transcripts assigned to PTS systems “SEED subcategories of “Membrane Transport (SEED L1)” in control and *L. paracasei* (LpC)-treated reactor mimicking the proximal colon section (model 1).

**Day**	**16**			**18**			**24**		
**Relative abundance (%)**	**CR**	**LpC**	**Ratio LpC/CR**	**CR**	**LpC**	**Ratio LpC/CR**	**CR**	**LpC**	**Ratio LpC/CR**
Fructose and mannose inducible PTS	0.12	0.15	1.26	0.08	0.15	1.96	0.09	0.14	1.52
Galactose-inducible PTS	0.10	0.12	1.22	0.09	0.11	1.26	0.10	0.14	1.43
Sucrose-specific PTS	0.32	0.40	1.23	0.20	0.29	1.43	0.18	0.35	1.98

*PTS, phosphotransferase systems*.

### Impact of *L. paracasei* on *faecalibacterium* Transcriptome

As we observed an increase in relative abundance of *Faecalibacterium* after the addition of *L. paracasei* both in 16S rRNA amplicon sequencing and qPCR, we performed a targeted analysis on transcriptome from *Faecalibacterium* (33.000–75.000 transcripts).

Most of transcripts were assigned to “Carbohydrates” (~25%), “Protein metabolism” (~16%), and “Clustering-based subsystems” (~14%) (Table S5). Addition of *L. paracasei* had little impact on relative abundance of most SEED categories L1, but “Clustering- based subsystems” and “Metabolism of aromatic compounds” were significantly (*p* < 0.05) reduced and increased, respectively. Increase of the latter was due to increased abundance of transcripts (1.2–1.3 fold) of acetyl-CoA acetyltransferase in PC_LpC (Table S6). Acetyl-CoA acetyltransferase is involved in several pathways, therefore we also observed enhanced relative abundance of SEED subcategories level 2 “Anaerobic degradation of aromatic compounds” (subcategory level L3 “Anaerobic benzoate metabolism”), “Fermentation” (“Butanol biosynthesis”), and “Lysine, threonine, methionine, and cysteine” (“Lysine fermentation”) in PC_LpC. Also, the transcription of acetate kinase was enhanced 1.1–1.8 fold in PC_LpC. Acetate kinase is involved in pathways represented by SEED categories related to “Fermentations” (“Fermentations: Lactate,” “Fermentations: Mixed acid”), “Lysine, threonine, methionine, and cysteine” (“Lysine fermentation”), “Central carbohydrate metabolism” (“Pyruvate metabolism II: acetyl-CoA, acetogenesis from pyruvate”), and “Sugar Alcohols” (“Ethanolamine utilization”).

In contrast, 4-alpha-glucanotransferase (amylomaltase) (EC 2.4.1.25) and Oligopeptide ABC transporter, periplasmic oligopeptide-binding protein OppA (TC 3.A.1.5.1) were 1.3–1.7 fold and 1.5–1.7 fold higher expressed in control fermentations (PC_CR), respectively.

#### Metabolic Activity of the Gut Microbiota

Metabolic activity at proximal colon conditions was assessed using HPLC-RI. Metabolite concentrations were stable and similar for PC_CR and PC_LpC throughout the 10 days treatment period (Figure S2). The main metabolite was acetate with concentrations around 55 mM followed by butyrate (25 mM) and propionate (8 mM).

### *F. prausnitzii* DSM 17677 in Co-culture With *L. paracasei*

**Figure 5:**
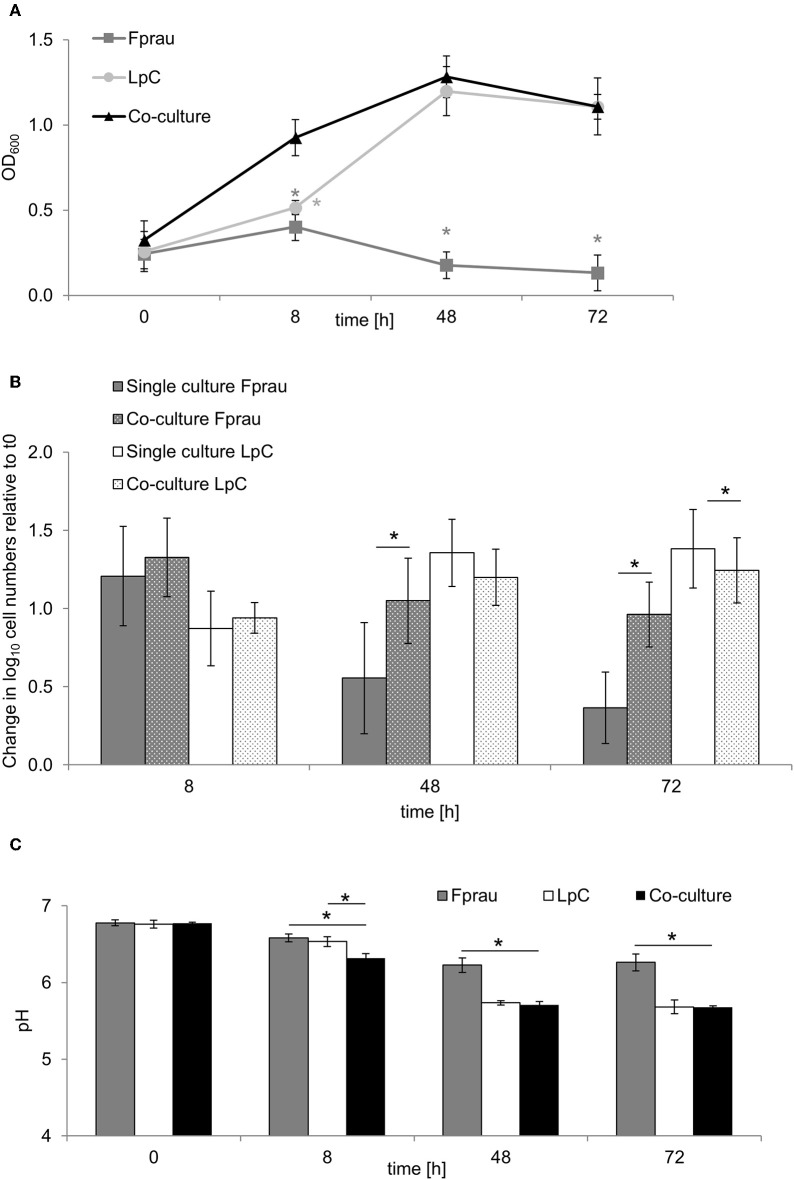
Single and co-culture study of *L. paracasei* (LpC) and *F. prausnitzii* (Fprau). **(A)** OD_600_ values of single and co-cultures of *L. paracasei* and *F. prausnitzii*. **(B)** Changes in cell numbers (log_10_ cell mL^−1^) of *L. paracasei* and *F. prausnitzii* in single and co-cultures measured with qPCR **(C)** pH values in single and co-cultures of *L. paracasei* and *F. prausnitzii*. Values are means ± SD of triplicate analysis of four separate experiments (*n* = 12), except for time point 72 h that was tested in three different experiments (*n* = 9); Values with an asterisk (*) indicate significant difference between single and co-cultures growth conditions (*p* < 0.05).

The increase in *Faecalibacterium* abundance associated with *L. paracasei* in proximal colon conditions (model 1) led us to explore whether there are direct interactions of the two species in batch fermentations (Figure 5). In single cultures, *F. prausnitzii* reached maximum OD of 0.4 ± 0.1 after 8 h that decreased to 0.1 ± 0.1 after 72 h (Figure 5A). Within 8 h, log cell numbers increased by 1.3, and a decrease of *F. prausnitzii* cell numbers after 48 and 72 h was also observed by qPCR (Figure 5B). The pH dropped from 6.8 ± 0.04 to 6.2 ± 0.1 within 48 h and remained stable thereafter (Figure 5C).

In co-cultures, maximum OD values for *L. paracasei* and *F. prausnitzii* were measured after 48 h (1.3 ± 0.1), they were similar to OD values recorded when *L. paracasei* was grown alone (1.2 ± 0.1). Also, pH values were similar in co-cultures and in *L. paracasei* single cultures and were significantly lower than of *F. prausnitzii* single cultures. *L. paracasei* cell numbers increased up to 48 h of incubation in single cultures as well as co-cultures (Figures 5A,B). In co-cultures, *F. prausnitzii* log cell numbers increased by 1.3 similar to single culture after 8 h incubation, however, log cell numbers decreased significantly less in co-culture thereafter. Butyrate concentrations were not significantly different in *F. prausnitzii* single cultures (5.3 ± 0.9 mM) compared to co-cultures (5.9 ± 2.2 mM) at all tested time points (Table S7).

### *C. difficile* DSM 1296 Growth and Toxin Production in Co-cultures With *L. paracasei*

**Figure 6:**
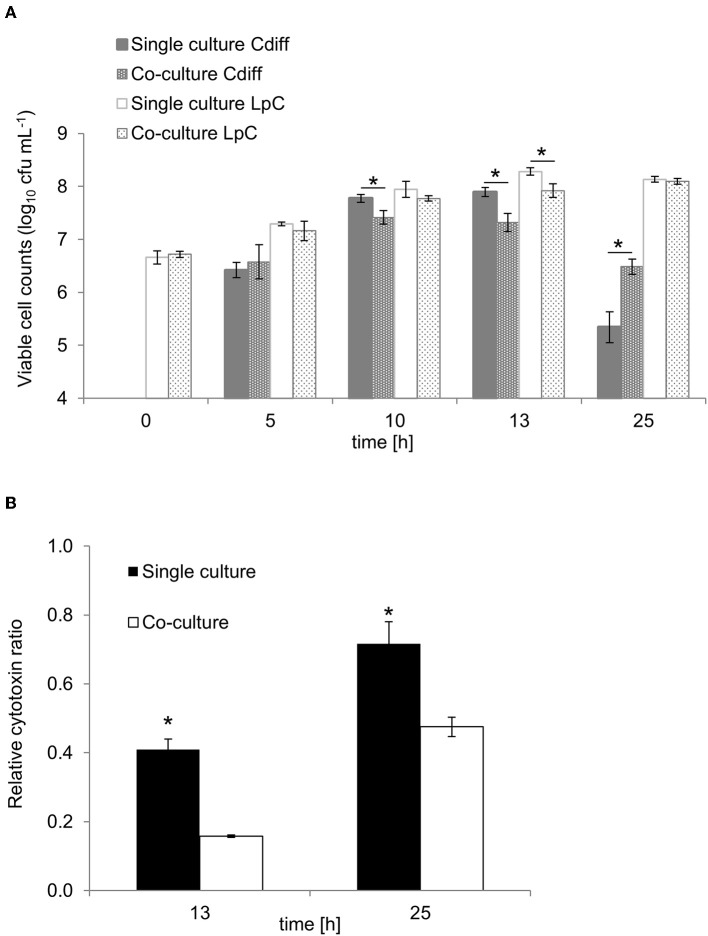
Growth and cytotoxin production in co-culture study of *L. paracasei* (LpC) with *C. difficile* DSM 1296 (Cdiff). **(A)** Viable cell counts (log_10_ cfu mL^−1^) of *C. difficile* and *L. paracasei* in single and co-cultures. **(B)** Cytotoxin titers produced during single and co-cultures after 13 and 25 h of incubation calculated relative to *C. difficile* cell counts. Values are means ± SD of triplicate analysis of two separate experiments (*n* = 6). Values with an asterisk (*) correspond to growth or cytotoxin titers in single cultures that are significantly different from co-cultures.

To investigate the potential of *L. paracasei* to attenuate growth and toxin production of *C. difficile*, we first performed batch cultures of *L. paracasei* co-cultivated with *C. difficile* DSM 1296. Due to slower growth, *L. paracasei* was inoculated 5 h ahead of *C. difficile* to yield a balanced growth of both strains (Figure 6A). Significantly reduced viable cell numbers were measured for *C. difficile* after 10 and 13 h of fermentation in co-cultures (7.4 ± 0.1 and 7.3 ± 0.2 log_10_ cfu mL^−1^, respectively) compared to pure cultures (7.8 ± 0.1 and 7.9 ± 0.1 log_10_ cfu mL^−1^, respectively). After 25 h cell counts decreased to 5.3 ± 0.3 and 6.5 ± 0.2 in pure and co-cultures, respectively. Cytotoxin titers were significantly lower in co-cultures compared to single cultures after 13 and 25 h fermentation (Figure 6B). The pH was significantly lower in co-cultures compared to single cultures after 25 h (Table S8).

Lactate and formate formation were significantly higher in single cultures compared to co-cultures for *L. paracasei* and *C. difficile*, respectively, while acetate formation in *C. difficile* single cultures was similar to co-cultures (Table S9).

### *L. paracasei* Impacts C. *difficile* DSM 1296 and NCTC 13307 Growth and Toxin Production in Distal Colon Conditions

The interaction between *L. paracasesi* and *C. difficile* was further investigated in complex microbiota at distal colon conditions as we previously showed that *C. difficile* did not establish in the proximal colon section of our colonic fermentation models (35).

The impact of preventive *L. paracasei* inoculation on *C. difficile* DSM 1296 colonization was determined in model 2. In model 3, *L. paracasei* was inoculated after establishment of *C. difficile* and was tested on *C. difficile* NCTC 13307 colonization in combination with metronidazole, an antibiotic prescribed in case of CDI.

**Figure 7:**
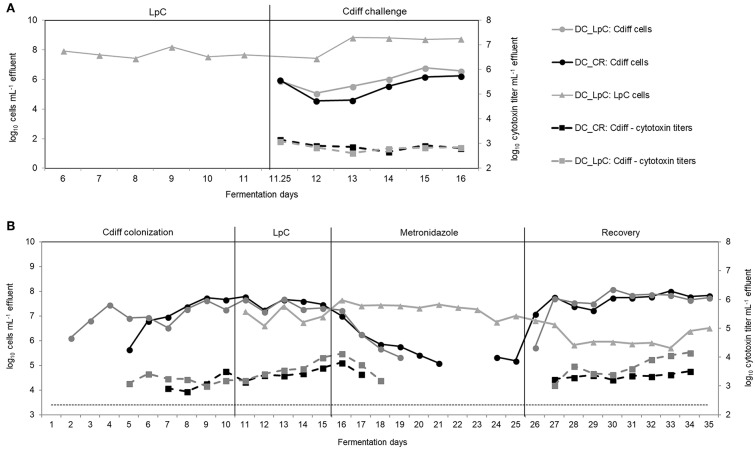
*L. paracasei* (LpC) and *C. difficile* (Cdiff) cell numbers (log_10_ cell mL^−1^) and cytotoxin titers in effluent samples from reactors mimicking the distal colon section. **(A)** Model 2. *L. paracasei* was inoculated twice daily from day 6 on into *L. paracasei* (LpC)-treated reactor mimicking the proximal colon section (PC_LpC) and on day 11 *C. difficile* DSM 1296 vegetative cells were inoculated into distal colon reactors of both test systems (DC_CR and DC_LpC). **(B)** Model 3. *C. difficile* NCTC 13307 was inoculated twice (day 1–2) in DC_CR and DC_LpC, and cell numbers and cytotoxin titer were monitored during different experimental periods. *L. paracasei* was inoculated twice daily in DC_LpC starting from day 11. Both reactors were treated with metronidazole from day 16–25 and recovery was monitored during days 26–35. (– – –) C. difficile detection limit of 3.4 log_10_ cell numbers mL−1.

In the preventive approach (model 2), *L. paracasei* remained stable during days 6–11 in DC_LpC (log_10_ 7.7 ± 0.3 cells mL^−1^ effluent, Figure 7A). However, after *C. difficile* inoculation cell numbers of *L. paracasei* increased by more than one log during the first days and remained constant thereafter, averaging log_10_ 8.8 ± 0.1 cells mL^−1^ effluent. On day 11, *C. difficile* DSM 1296 (log_10_ 8.5 cells) was added into both distal colon reactors of the model. During the first 24 h, *C. difficile* cell numbers decreased by approximately 1 log, and then steadily increased from day 13 to 16 to reach a similar concentration of log_10_ 6.2 and 6.6 cells mL^−1^ in DC_CR and DC_LpC, respectively. Toxin titers were similar in control and treatment reactor throughout the fermentation period, with average titers of log_10_ 2.9 ± 0.2 and 2.8 ± 0.2 per mL effluent in DC_CR and DC_LpC, respectively.

Next, we examined the impact of *L. paracasei* on already established *C. difficile* in model 3. The effect of *L. paracasei* on *C. difficile* was assessed during 5 days after colonization of *C. difficile* NCTC 13307 and then during 10 days of metronidazole treatment followed by 10 days of post antibiotic recovery (Figure 7B). *C. difficile* cell numbers were similar in DC_CR and DC_LpC before the addition of *L. paracasei* with average values of log_10_ 7.6 ± 0.2 and log_10_ 7.4 ± 0.2 cells mL^−1^ effluent (day 8–10), respectively.

After addition, *L. paracasei* cell numbers ranged at log_10_ 7.0 ± 0.3 per mL and slightly increased during metronidazole treatment (log_10_ 7.3 ± 0.3 cells mL^−1^). During the first 3 days of recovery *L. paracasei* decreased by around 1 log and reached final abundance of log_10_ 6.0 ± 0.3 cells mL^−1^ at day 35. *C. difficile* NCTC 13307 growth continuously decreased in both reactors during metronidazole treatment and reached the detection limit after 4 days in DC_LpC compared to 6 days in DC_CR. Similarly, toxin production was below detection limit shortly after start of metronidazole treatment. *C. difficile* reappeared at 5.3 cells mL^−1^ in DC_CR at days 24 and 25 of metronidazole treatment. A fast recovery of *C. difficile* was observed in both DC's with a slight delay for DC_LpC. After 2 day recovery, *C. difficile* cell numbers were stable in both reactors (log_10_ 7.7 ± 0.2 and 7.8 ± 0.2 mL^−1^ in DC_CR and DC_LpC, respectively) until the end of the period. Cytotoxin titers were similar in both reactors during the recovery period.

## Discussion

Probiotics can transiently colonize the human colon, leading to an alteration of both the composition and activity of the commensal microbiota (50). It was suggested that these changes could enhance general homeostasis of the gut microbiota, thereby preventing overgrowth of enteric pathogens such as *C. difficile* (51). The hindered accessibility of the gastrointestinal tract hampers clinical studies on the effect of probiotics on gut microbiota of different colonic sections. In the current work we applied different *in vitro* colonic fermentation models operated with controlled conditions to test the response of the commensal elderly gut microbiota to the probiotic strain *L. paracasei* CNCM I-1518 and to investigate the probiotic-pathogen interaction with *C. difficile*, independently of host factors such as the epithelial cell layer and immune response.

Levels of *L. paracasei* applied in the *in vitro* model were in the range of fecal concentrations of the same strain assessed *in vivo* (24, 52). Upon daily addition, numbers of *L. paracasei* remained stable or even increased indicating the possibility of temporary persistence. Colonization of *L. paracasei* in the reactors was nevertheless transient since cessation of probiotic addition was accompanied by a rapid wash-out within 3 days (data not shown). Transient properties were ascribed to many other *Lactobacillus* species used as probiotics (50).

Despite low abundance relative to commensal bacteria and transient properties, probiotics impact elderly microbiota composition. Consumption of probiotics led to an increase in bifidobacteria, lactobacilli or *Faecalibacterium* (53–55) and decreased the abundance of opportunistic pathogens (56). Here, we showed increases in abundances of phylotypes belonging to the *Lactobacillales*, including *Lactobacillus* and *Enterococcus*, suggesting that *L. paracasei* enhances niche colonization of closely related genera (57). An increase in fecal concentrations of the *Lactobacillus/Enterococcus* group was previously observed upon consumption of fermented milk containing a *Lactobacillus salivarius* strain (58) or *Lactobacillus rhamnosus* (59) by healthy adults.

It was earlier reported that Bacteroidetes were more abundant in elderly compared to adults (60). Here, *L. paracasei* supplementation was associated with decreased abundances of phylotypes affiliated to Bacteroidetes. Compositional modifications observed after addition of *L. paracasei* might be related to changes in trophic interactions.

In recent studies no effect on fecal microbiota composition but community-wide transcriptional changes were observed after consumption of fermented milk products (61, 62). McNulty et al. observed that 7-weeks consumption of fermented milk product containing *B. animalis* subsp *lactis* CNCM I-2494 did not change composition based on 16S rRNA gene sequencing, but interesting changes in fecal gene expression were measured, notably related to plant polysaccharide metabolism and SCFA production, indicating an expansion of the carbohydrate metabolizing ability of the microbiota during transient colonization with the product (62). In another study, healthy elderly subjects were given *Lactobacillus rhamnosus* GG (61). Similarly, as McNulty et al., no modification of the microbiota composition but a clear transcriptional response was observed. Across all functional categories, increased expression of genes involved in flagellar motility, chemotaxis, and adhesion from *Bifidobacterium* and the dominant butyrate producers *Roseburia* and *Eubacterium* were observed during probiotic consumption. These studies highlight the interest of analyzing transcriptional activity of gut microbiota. Here we determined the impact of *L. paracasei* CNCM I-1518 on the proximal colon microbiota. Colonic microbiota transcriptional functional profile varied little during the test period confirming stability of the fermentation model. The high proportion of transcripts assigned to “Carbohydrates” and “Protein metabolism” indicated bacterial growth, carbohydrate fermentation and SCFA formation in the presence of high substrate concentrations, successfully mimicking the scenario in the human proximal colon as observed before (63). Despite shifts in microbiota composition, we only observed minor alterations of relative abundance of microbial functions upon addition of *L. paracasei*, possibly due to functional redundancy of the intestinal microbiota. For example, a shift in butyrate-producing phylotypes after addition of *L. paracasei* with decreased abundance of *Roseburia and Peptoniphilus*, and increased abundance of *Faecalibacterium* was observed, nevertheless, relative abundance of the SEED category “Fermentation” including transcripts related to butyrate formation was not impacted.

SCFA profiles in both control and treated reactors were highly comparable despite the presence of *L. paracasei*. A positive correlation between *L. paracasei* supplementation and *Faecalibacterium* was reported earlier for healthy adults (64). In co-cultures, *L. paracasei* enhanced survival of *F. prausnitzii* in the stationary growth phase. A possible reason could be the lower pH in co-cultures compared to pure *F. prausnitzii* cultures that may have protected cells from autolysis as it has been observed in several bacterial species (65). That there was no negative impact on growth of *F. prausnitzii* by *L. paracasei*, indicates that both strains do not compete for substrates or are inhibited by metabolites formed in the test conditions.

Probiotics were suggested as alternative treatment for gastrointestinal diseases including antibiotic-related infections, such as CDI (66). CDI often occurs after treatment with broad-spectrum antibiotics and incidences are increased in the elderly population (13). Several studies reported a reduction in *C. difficile-*associated diarrhea with probiotics, including *Saccharomyces boulardii* (67) and *Lactobacillus acidophilus* in combination with *L. casei* (68) as well as a milk drink with yogurt starter bacteria and *L. paracasei* (29, 30). *S. boulardii* acts by secreting a protease which is able to cleave toxin A and possesses enzymatic activity against *C. difficile* toxin B (69). However, for most probiotics the mechanism of action in CDI remains unknown. Here, we found reduced *C. difficile* cytotoxin titers when co-cultivated with *L. paracasei* compared to pure cultures, but no reduction in continuous fermentation studies with *L. paracasei* tested as preventive treatment (model 2) or as adjuvant therapy to metronidazole treatment (model 3). During co-cultures, *C. difficile* was inoculated 5 h after *L. paracasei* to account for the fast growth of the first, while for the modeled microbiota, *C. difficile* only grew in distal reactor conditions set at a pH of 6.8 which is less favorable for lactobacilli growth (35). Our data suggest that host factors that are not accounted in the *in vitro* colonic fermentation model, may contribute to the prevention effect of probiotics observed *in vivo* (19, 70).

To conclude, this is the first-time investigation of the effect of a candidate probiotic strain on the transcriptome of elderly gut microbiota using *in vitro* intestinal fermentation models. We showed a compositional and functional response of the microbiota on *L. paracasei*, with an enhancing effect on *Faecalibacterium* abundance and activity, a decrease in abundance of H_2_ and CH_4_ fermentative bacteria, and an increase in carbohydrate utilization, indicating a possible contribution of *L. paracasei* in the trophic interaction of dietary carbohydrate utilization with the commensal microbiota. We thus showed that the *L. paracasei* strain directly interacts with the human gut microbiota independent of the host. In contrast, no effect of *L. paracasei* was observed on *C. difficile* in complex microbiota uncoupled from the host when tested as preventive treatment or concomitantly to metronidazole, which may be partly due to the limits of *in vitro* microbiota models not accounting for host factors. Thus, host-microbiota interaction studies should be conducted for further investigations of the mechanism of *L. paracasei* in treatment or prevention of CDI.

## Data Availability Statement

The datasets generated for this study can be found in the National Center for Biotechnology Information (NCBI) Sequence Read Archive (SRA) under bioproject accession number SRP144222.

## Ethics Statement

The Ethics Committee of ETH Zurich exempted this study from review because sample collection was not in terms of intervention.

## Author Contributions

SF, CC, CS, CF, MD, and CL conceived and designed the experiments. SF and MV conducted the experiments. SF, MV, CS, and MD conducted the analysis. SF, CC, CS, MD, and CL interpreted the results and wrote the paper. All authors read and approved the final manuscript.

### Conflict of Interest

MD and CF are employed by Danone Research (Palaiseau, France). The remaining authors declare that the research was conducted in the absence of any commercial or financial relationships that could be construed as a potential conflict of interest.

## Footnotes

^1^https://rrndb.umms.med.umich.edu/about/
