# Supplementary material for: In vitro Study of Lactobacillus paracasei CNCM I-1518 in Healthy and Clostridioides difficile Colonized Elderly Gut Microbiota
Source: Front Nutr. 2019 Dec 10;6:184. doi: 10.3389/fnut.2019.00184 (PMC6914822; doi:10.3389/fnut.2019.00184)
Supplement: Supplementary file 1 [file Data_Sheet_1.docx]

**Table S 1** Primers used for enumeration of specific bacterial groups with qPCR.

| **Name** | **Sequence 5'-3'** | **Target gene/purpose** | **Target locus** | **Reference** |
| --- | --- | --- | --- | --- |
| OFF 2201 | GTTAGCACCGCTTAAAGACG | *Lactobacillus paracasei* CNCM I-1518 | CRISPR | [1] |
| OFF 2202 | GCCATAAGCGTGTTAGCCG |  |  |  |
| Eub338F | ACTCCTACGGGAGGCAGCAG | Total 16S rRNA genes | 16S rRNA | [2] |
| Eub518R | ATTACCGCGGCTGCTGG |  |  |  |
| Bac303F | GAAGGTCCCCCACATTG | *Bacteroides-Prevotella* group | 16S rRNA | [3] |
| Bfr-Femrev | CGCKACTTGGCTGGTTCAG |  |  |  |
| F_Lacto 05 | AGCAGTAGGGAATCTTCCA | *Lactobacillus/Pediococcus/*  *Leuconostoc* spp. | 16S rRNA | [4] |
| R_Lacto 04 | CGCCACTGGTGTTCYTCCATATA |  |  |  |
| Fprau223F | GATGGCCTCGCGTCCGATTAG | *Faecalibacterium prausnitzii* | 16S rRNA | [5] |
| Fprau420R | CCGAAGACCTTCTTCCTCC |  |  |  |
| G_RosEub_F | TCAAATCMGGIGACTGGGTWGA | *Roseburia* spp.*/Eubacterium rectale* | Butyryl-CoA CoA transferase | [6] |
| G_Ros_R | TCGATACCGGACATATGCCAKGAG |  |  |  |
| G_Eub_R | TCATAACCGCCCATATGCCATAG |  |  |  |
| Met915F | AGG AATTGGCGGGGGAGCAC | *Methanobrevibacter smithii* | 16S rRNA | [7] |
| 1’100AR | TGGGTCTCGCTCGTTG |  |  |  |
| Cdiff_F | TTGAGCGATTTACTTCGGTAAAGA | *Clostridium difficile* | 16S rRNA | [8] |
| Cdiff_R2 | CCATCCTGTACTGGCTCACCT |  |  |  |

**Table S 2** RNA sequencing information colonic model 1.

| **Day** | **Treatment** | **total overlapped reads** | **16S rRNA genes** | **mRNA** | **%mRNA** | **assigned to SEED subsystems** | **%Subsystems** |
| --- | --- | --- | --- | --- | --- | --- | --- |
| 16 | CR | 4.891.106 | 4.496.540 | 394.566 | 8.1 | 226.393 | 57.4 |
|  | TR | 7.446.549 | 6.805.385 | 641.164 | 8.6 | 135.390 | 21.1 |
| 18 | CR | 6.575.626 | 5.934.633 | 640.993 | 9.7 | 175.859 | 27.4 |
|  | TR | 4.535.456 | 4.050.445 | 485.011 | 10.7 | 228.744 | 47.2 |
| 24 | CR | 7.834.060 | 7.204.870 | 629.190 | 8.0 | 326.804 | 51.9 |
|  | TR | 12.783.241 | 11.835.325 | 947.916 | 7.4 | 211.925 | 22.4 |

**Table S 3** Relative abundance of bacterial orders (%) (rRNA reads). In bold: orders that are decreased in PC_LpC compared to PC_CR (*p*<0.05).

| **Day** | **16** | | | **18** | | | **24** | | |
| --- | --- | --- | --- | --- | --- | --- | --- | --- | --- |
| **Relative abundance** | **CR** | **LpC** | **Ratio LpC/CR** | **CR** | **LpC** | **Ratio LpC/CR** | **CR** | **LpC** | **Ratio LpC/CR** |
| uc Bacteria | 0.69 | 0.69 | 1.00 | 1.08 | 0.68 | 0.63 | 1.01 | 0.98 | 0.97 |
| uc Gammaproteobacteria | 0.31 | 0.35 | 1.12 | 0.47 | 0.22 | 0.47 | 0.33 | 0.59 | 1.76 |
| Enterobacteriales | 3.22 | 3.97 | 1.23 | 4.12 | 2.38 | 0.58 | 3.36 | 5.72 | 1.70 |
| Bacteroidales | 1.42 | 1.92 | 1.35 | 2.12 | 1.81 | 0.85 | 7.60 | 5.34 | 0.70 |
| Bifidobacteriales | 0.08 | 0.09 | 1.06 | 0.08 | 0.05 | 0.60 | 0.08 | 0.03 | 0.33 |
| Coriobacteriales | 0.18 | 0.21 | 1.19 | 0.15 | 0.11 | 0.73 | 0.13 | 0.10 | 0.72 |
| Lactobacillales | 3.25 | 4.70 | 1.45 | 5.04 | 4.70 | 0.93 | 7.11 | 7.56 | 1.06 |
| Clostridiales | 89.98 | 87.12 | 0.97 | 85.76 | 89.00 | 1.04 | 78.07 | 77.74 | 1.00 |
| **Methanobacteriales** | **0.04** | **0.02** | **0.41** | **0.04** | **0.01** | **0.20** | **0.08** | **0.01** | **0.09** |

uc: unclassified

**Table S 4** Taxonomic assignment mRNA reads in PC_CR or in PC_LpC (order level). In bold: orders that are decreased in PC_LpC relative to PC_CR (*p*<0.05).

| **Day** | **16** | | | **18** | | | **24** | | |
| --- | --- | --- | --- | --- | --- | --- | --- | --- | --- |
| **Relative abundance** | **CR** | **LpC** | **Ratio LpC/CR** | **CR** | **LpC** | **Ratio LpC/CR** | **CR** | **LpC** | **Ratio LpC/CR** |
| uc Bacteria | 6.07 | 6.28 | 1.03 | 6.39 | 6.43 | 1.01 | 6.48 | 6.26 | 0.97 |
| **Enterobacteriales** | **0.48** | **0.25** | **0.52** | **0.33** | **0.25** | **0.77** | **0.58** | **0.29** | **0.49** |
| Bacteroidales | 2.04 | 1.36 | 0.66 | 1.87 | 2.35 | 1.26 | 7.64 | 6.65 | 0.87 |
| Bifidobacteriales | 0.08 | 0.07 | 0.86 | 0.03 | 0.06 | 2.05 | 0.01 | 0.02 | 2.74 |
| Coriobacteriales | 0.36 | 0.30 | 0.82 | 0.17 | 0.21 | 1.26 | 0.18 | 0.23 | 1.30 |
| uc Firmicutes | 15.45 | 15.85 | 1.03 | 15.84 | 15.86 | 1.00 | 15.80 | 15.50 | 0.98 |
| **Lactobacillales** | **1.68** | **1.33** | **0.79** | **1.00** | **0.75** | **0.75** | **1.12** | **0.95** | **0.85** |
| Clostridiales | 71.86 | 72.55 | 1.01 | 72.33 | 72.01 | 1.00 | 65.67 | 67.63 | 1.03 |

uc: unclassified

**Table S 5** Relative abundance of SEED categories of transcripts assigned to *Faecalibacterium* in proximal colon section (model 1). In bold: SEED categories that are increased or decreased in PC_LpC relative to PC_CR (*p*<0.05).

| **Day** | **16** | | | **18** | | | **24** | | |
| --- | --- | --- | --- | --- | --- | --- | --- | --- | --- |
| **Relative abundance** | **CR** | **LpC** | **Ratio LpC/CR** | **CR** | **LpC** | **Ratio LpC/CR** | **CR** | **LpC** | **Ratio LpC/CR** |
| Amino Acids and Derivatives | 7.88 | 7.78 | 0.99 | 8.31 | 8.84 | 1.06 | 8.55 | 7.79 | 0.91 |
| Carbohydrates | 24.05 | 25.51 | 1.06 | 25.42 | 25.08 | 0.99 | 25.13 | 24.80 | 0.99 |
| Cell Division and Cell Cycle | 0.95 | 0.84 | 0.88 | 0.91 | 0.96 | 1.05 | 1.12 | 0.82 | 0.73 |
| Cell Wall and Capsule | 3.73 | 4.11 | 1.10 | 3.85 | 3.81 | 0.99 | 3.47 | 2.71 | 0.78 |
| **Clustering-based subsystems** | **14.64** | **13.70** | **0.94** | **14.89** | **14.59** | **0.98** | **15.75** | **13.62** | **0.86** |
| Cofactors, Vitamins, Prosthetic Groups, Pigments | 2.72 | 2.51 | 0.92 | 2.35 | 2.50 | 1.06 | 2.21 | 2.76 | 1.25 |
| DNA Metabolism | 2.02 | 1.75 | 0.87 | 1.84 | 2.11 | 1.15 | 2.08 | 1.86 | 0.90 |
| Dormancy and Sporulation | 0.14 | 0.16 | 1.15 | 0.17 | 0.19 | 1.12 | 0.18 | 0.08 | 0.42 |
| Fatty Acids, Lipids, and Isoprenoids | 3.42 | 3.13 | 0.91 | 2.83 | 3.23 | 1.14 | 2.10 | 3.05 | 1.45 |
| Iron acquisition and metabolism | 0.22 | 0.18 | 0.82 | 0.20 | 0.08 | 0.40 | 0.20 | 0.18 | 0.89 |
| Membrane Transport | 5.61 | 7.76 | 1.38 | 4.27 | 3.97 | 0.93 | 4.49 | 5.74 | 1.28 |
| **Metabolism of Aromatic Compounds** | **1.08** | **1.19** | **1.10** | **1.11** | **1.42** | **1.28** | **0.99** | **1.20** | **1.22** |
| Miscellaneous | 3.92 | 3.45 | 0.88 | 3.96 | 3.93 | 0.99 | 4.88 | 4.21 | 0.86 |
| Motility and Chemotaxis | 0.29 | 0.50 | 1.70 | 0.95 | 0.31 | 0.33 | 0.58 | 0.31 | 0.54 |
| Nitrogen Metabolism | 0.14 | 0.14 | 1.00 | 0.16 | 0.16 | 1.00 | 0.18 | 0.20 | 1.12 |
| Nucleosides and Nucleotides | 2.17 | 1.90 | 0.87 | 2.60 | 2.82 | 1.09 | 3.47 | 2.98 | 0.86 |
| Phages, Prophages, Transposable elements, Plasmids | 0.52 | 0.46 | 0.89 | 0.46 | 0.51 | 1.12 | 0.52 | 0.57 | 1.11 |
| Phosphorus Metabolism | 0.21 | 0.22 | 1.05 | 0.11 | 0.12 | 1.11 | 0.16 | 0.13 | 0.82 |
| Photosynthesis | 0.02 | 0.02 | 0.90 | 0.01 | 0.02 | 2.28 | 0.02 | 0.03 | 1.50 |
| Potassium metabolism | 0.08 | 0.06 | 0.84 | 0.06 | 0.08 | 1.40 | 0.05 | 0.05 | 1.20 |
| Protein Metabolism | 16.64 | 16.28 | 0.98 | 16.16 | 15.35 | 0.95 | 14.71 | 16.56 | 1.13 |
| RNA Metabolism | 4.04 | 3.29 | 0.81 | 3.84 | 4.36 | 1.13 | 4.51 | 4.28 | 0.95 |
| Regulation and Cell signaling | 1.03 | 0.94 | 0.91 | 0.91 | 0.96 | 1.05 | 1.17 | 1.00 | 0.85 |
| Respiration | 2.01 | 1.65 | 0.82 | 2.01 | 1.82 | 0.91 | 1.07 | 2.10 | 1.96 |
| Secondary Metabolism | 0.02 | 0.03 | 1.35 | 0.01 | 0.01 | 0.76 | 0.01 | 0.05 | 3.51 |
| Stress Response | 0.86 | 0.75 | 0.87 | 0.71 | 0.79 | 1.11 | 0.75 | 1.09 | 1.45 |
| Sulfur Metabolism | 0.12 | 0.16 | 1.35 | 0.11 | 0.10 | 0.90 | 0.15 | 0.15 | 1.00 |
| Virulence, Disease and Defense | 1.47 | 1.55 | 1.05 | 1.79 | 1.89 | 1.06 | 1.51 | 1.69 | 1.12 |

**Table S 6** Relative abundance of transcripts that were different in PC_LpC relative to PC_CR in proximal colon section (model 1).

| **Day** | **16** | | | | **18** | | | | **24** | | | |
| --- | --- | --- | --- | --- | --- | --- | --- | --- | --- | --- | --- | --- |
| **Relative abundance** | **CR** | **LpC** | **Ratio LpC/CR** | **CR** | | **LpC** | **Ratio LpC/CR** | **CR** | | **LpC** | **Ratio LpC/CR** |  |
| Acetyl-CoA acetyltransferase (EC 2.3.1.9) | 0.70 | 0.89 | 1.27 | 0.89 | | 1.03 | 1.15 | 0.73 | | 0.87 | 1.20 |  |
| Acetate kinase (EC 2.7.2.1) | 0.31 | 0.48 | 1.53 | 0.35 | | 0.64 | 1.84 | 0.72 | | 0.77 | 1.07 |  |
| 4-alpha-glucanotransferase (amylomaltase) (EC 2.4.1.25) | 1.11 | 0.80 | 0.72 | 1.18 | | 0.92 | 0.78 | 1.24 | | 0.75 | 0.60 |  |
| Oligopeptide ABC transporter, periplasmic oligopeptide-binding protein OppA (TC 3.A.1.5.1) | 0.22 | 0.15 | 0.65 | 0.30 | | 0.19 | 0.61 | 0.32 | | 0.18 | 0.57 |  |

**Table S 7** Production of metabolites (mM) in co-cultures and single cultures of *L. paracasei* and *F. prausnitzii* of all time points analyzed in co-culture study.

| **Time (h)** | **Culture** | **Lactate** | **Formate** | **Acetate** | **Butyrate** | **Propionate** |
| --- | --- | --- | --- | --- | --- | --- |
| **0** | Co-culture | ND | ND | 26.4 ± 7.2 | ND | 8.5 ± 1.1 |
|  | *L. paracasei* | ND | ND | 28.0 ± 0.9 | ND | 9.5 ± 0.8 |
|  | *F. prausnitzii* | ND | 0.5 ± 0.4 | 27.7 ± 3.0 | ND | 9.8 ± 1.3 |
| **8** | Co-culture | 8.3 ± 1.5 | 4.2 ± 1.5 | 27.6 ± 1.3 | 1.9 ± 1.2 | 8.1 ± 0.7 |
|  | *L. paracasei* | 8.3 ± 1.6 | ND | 27.1 ± 5.8 | ND | 9.0 ± 1.5 |
|  | *F. prausnitzii* | ND | 4.3 ± 1.0 | 28.9 ± 1.4 | 2.2 ± 0.8 | 8.8 ± 3.8 |
| **48** | Co-culture | 21.9 ± 1.0 | 9.8 ± 1.6 | 26.3 ± 2.2 | 6.2 ± 1.2 | 7.5 ± 3.1 |
|  | *L. paracasei* | 31.9 ± 2.8* | 2.3 ± 2.2* | 28.7 ± 2.6* | ND | 8.8 ± 0.4 |
|  | *F. prausnitzii* | ND | 11.0 ± 2.0 | 28.5 ± 3.1* | 5.2 ± 1.6 | 9.7 ± 0.6* |
| **72** | Co-culture | 21.6 ± 1.7 | 8.3 ± 2.3 | 26.2 ± 2.8 | 5.9 ± 2.2 | 8.3 ± 1.2 |
|  | *L. paracasei* | 33.6 ± 3.1* | 1.5 ± 2.1* | 29.8 ± 1.3* | ND | 9.7 ± 0.5 |
|  | *F. prausnitzii* | ND | 10.3 ± 1.8 | 29.6 ± 1.9* | 5.3 ± 0.9 | 9.7 ± 0.6 |

Data are means ± SD of three replicates tested in 4 different experiments (n=12), except for time point 72h that was tested in 3 different experiments (n=9); samples were analyzed in duplicate. ND, not detected

Means with an asterisk (*) are significantly different from the co-culture (*p*<0.05).

**Table S 8** Changes in pH during incubation of co-cultures and the respective single cultures of *C. difficile* DSM 1296 and *L. paracasei* in co-culture study.

|  | **pH** | | |
| --- | --- | --- | --- |
| **Time (h)** | **Co-culture** | ***C. difficile*** | ***L. paracasei*** |
| **0** | 6.46 ± 0.07 | NA | 6.42 ± 0.04 |
| **5** | 6.32 ± 0.05 | 6.40 ± 0.11 | 6.35 ± 0.07 |
| **10** | 5.62 ± 0.02 | 6.01 ± 0.01* | 5.81 ± 0.03* |
| **13** | 5.54 ± 0.06 | 5.67 ± 0.04* | 5.63 ± 0.05 |
| **25** | 5.66 ± 0.02 | 5.75 ± 0.02* | 5.70 ± 0.02* |

Values represent means ± SD of three replicates tested in two separate experiments (n=6). Means with an asterisk (*) are significantly different from the co-culture (*p*<0.05). NA, not analyzed.

**Table S 9** Production of metabolites (mM) in co-cultures and single cultures of *L. paracasei* and *C. difficile* DSM 1296 of all time points analyzed in co-culture study.

| **Time (h)** | **culture** | **Lactate** | **Formate** | **Acetate** | **Butyrate** | **Propionate** |
| --- | --- | --- | --- | --- | --- | --- |
| **0** | Co-culture | 1.7 ± 0.3 | 0.8 ± 0.2 | 9.4 ± 0.6 | ND | 2.3 ± 0.1 |
|  | *L. paracasei* | 1.7 ± 0.3 | 1.6 ± 3.0 | 9.5 ± 3.1 | ND | 2.2 ± 0.9 |
|  | *C. difficile* | NA | NA | NA | NA | NA |
| **5** | Co-culture | 6.2 ± 0.5 | 0.7 ± 0.3 | 9.8 ± 0.6 | ND | 2.1 ± 0.3 |
|  | *L. paracasei* | 6.6 ± 0.6 | 0.7 ± 0.3 | 10.0 ± 0.5 | ND | 2.3 ± 0.3 |
|  | *C. difficile* | 0.3 ± 0.1* | 1.5 ± 3.1 | 10.1 ± 1.9 | ND | 2.3 ± 0.9 |
| **10** | Co-culture | 20.2 ± 1.5 | 4.0 ± 1.8 | 16.1 ± 1.7 | 0.9 ± 0.5 | 2.1 ± 0.3 |
|  | *L. paracasei* | 20.8 ± 5.5 | 1.0 ± 0.2 | 10.5 ± 0.4* | ND | 2.2 ± 0.2 |
|  | *C. difficile* | 1.3 ± 0.1* | 7.3 ± 0.5 | 18.3 ± 0.7 | 0.7 ± 0.3 | 2.0 ± 0.3 |
| **13** | Co-culture | 22.1 ± 0.7 | 4.2 ± 0.9 | 16.5 ± 0.7 | 1.2 ± 0.3 | 2.1 ± 0.3 |
|  | *L. paracasei* | 28.7 ± 1.4* | 0.9 ± 0.4* | 10.7 ± 0.6* | ND | 2.3 ± 0.5 |
|  | *C. difficile* | 3.6 ± 0.4* | 7.7 ± 1.0* | 20.0 ± 0.4 | 2.5 ± 0 .9* | 1.6 ± 0.4* |
| **25** | Co-culture | 22.1 ± 0.9 | 5.1 ± 0.5 | 18.7 ± 1.8 | 1.7 ± 0.5 | 2.3 ± 0.5 |
|  | *L. paracasei* | 28.7 ± 0.6* | 1.2 ± 0.1* | 10.9 ± 0.9* | ND | 2.3 ± 0.2 |
|  | *C. difficile* | 3.8 ± 0.4* | 7.8 ± 0.1* | 21.2 ± 0.7 | 2.9 ± 0.6* | 1.7 ± 0.2* |

Data are means ± SD of three replicates tested in two different experiments (n=6); samples were analyzed in duplicate. ND, not detected; NA, not analyzed.

Means with an asterisk (*) are significantly different from the co-culture (*p*< 0.05)

1. Collins JW, Chervaux C, Raymond B, Derrien M, Brazeilles R, Kosta A, Chambaud I, Crepin VF, Frankel G: **Fermented dairy products modulate *Citrobacter rodentium*-induced colonic hyperplasia.** *J Infect Dis* 2014, **210:**1029-1041.

2. Guo X, Xia X, Tang R, Zhou J, Zhao H, Wang K: **Development of a real-time PCR method for Firmicutes and Bacteroidetes in faeces and its application to quantify intestinal population of obese and lean pigs.** *Lett Appl Microbiol* 2008, **47:**367-373.

3. Ramirez-Farias C, Slezak K, Fuller Z, Duncan A, Holtrop G, Louis P: **Effect of inulin on the human gut microbiota: stimulation of *Bifidobacterium adolescentis* and *Faecalibacterium prausnitzii*.** *Br J Nutr* 2009, **101:**541-550.

4. Furet JP, Firmesse O, Gourmelon M, Bridonneau C, Tap J, Mondot S, Dore J, Corthier G: **Comparative assessment of human and farm animal faecal microbiota using real-time quantitative PCR.** *FEMS Microbiol Ecol* 2009, **68:**351-362.

5. Bartosch S, Fite A, Macfarlane GT, McMurdo ME: **Characterization of bacterial communities in feces from healthy elderly volunteers and hospitalized elderly patients by using real-time PCR and effects of antibiotic treatment on the fecal microbiota.** *Appl Environ Microb* 2004, **70:**3575-3581.

6. Vital M, Penton CR, Wang Q, Young VB, Antonopoulos DA, Sogin ML, Morrison HG, Raffals L, Chang EB, Huffnagle GB, et al: **A gene-targeted approach to investigate the intestinal butyrate-producing bacterial community.** *Microbiome* 2013, **1:**8.

7. Tymensen LD, McAllister TA: **Community structure analysis of methanogens associated with rumen protozoa reveals bias in universal archaeal primers.** *Appl Environ Microbiol* 2012, **78:**4051-4056.

8. Rinttila T, Kassinen A, Malinen E, Krogius L, Palva A: **Development of an extensive set of 16S rDNA-targeted primers for quantification of pathogenic and indigenous bacteria in faecal samples by real-time PCR.** *J Appl Microbiol* 2004, **97:**1166-1177.
