# Supplementary material for: In vitro Study of Lactobacillus paracasei CNCM I-1518 in Healthy and Clostridioides difficile Colonized Elderly Gut Microbiota
Source: Front Nutr. 2019 Dec 10;6:184. doi: 10.3389/fnut.2019.00184 (PMC6914822; doi:10.3389/fnut.2019.00184)
Supplement: Supplementary file 2 [file Image_1.pdf]

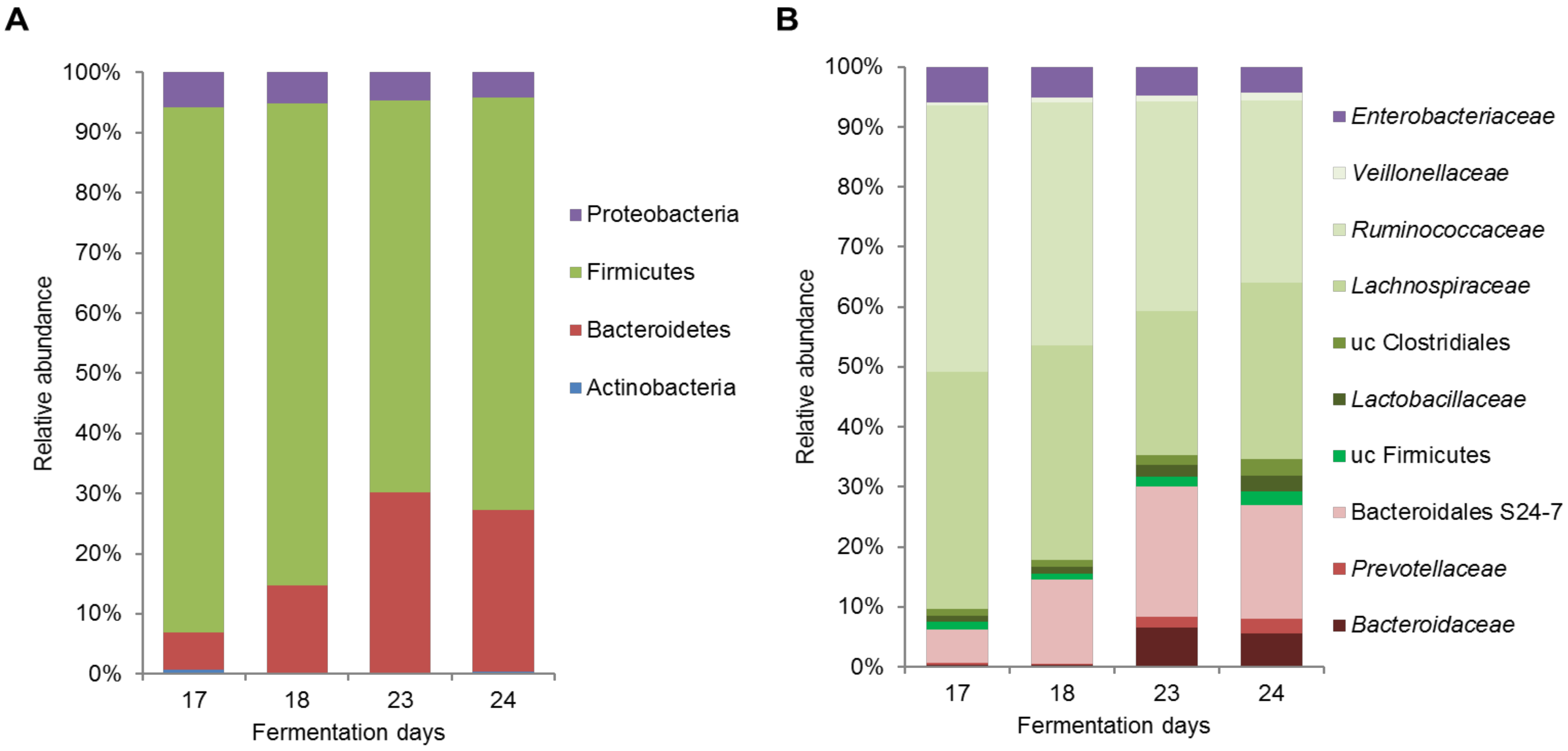

Figure S 1 Composition of fermentation effluent samples in PC_CR of model 1 assessed with 16S rRNA gene amplicon sequencing. **(A)** Phylum level **(B)** family level; uc, unclassified
