# Supplementary material for: In vitro Study of Lactobacillus paracasei CNCM I-1518 in Healthy and Clostridioides difficile Colonized Elderly Gut Microbiota
Source: Front Nutr. 2019 Dec 10;6:184. doi: 10.3389/fnut.2019.00184 (PMC6914822; doi:10.3389/fnut.2019.00184)
Supplement: Supplementary file 3 [file Image_2.pdf]

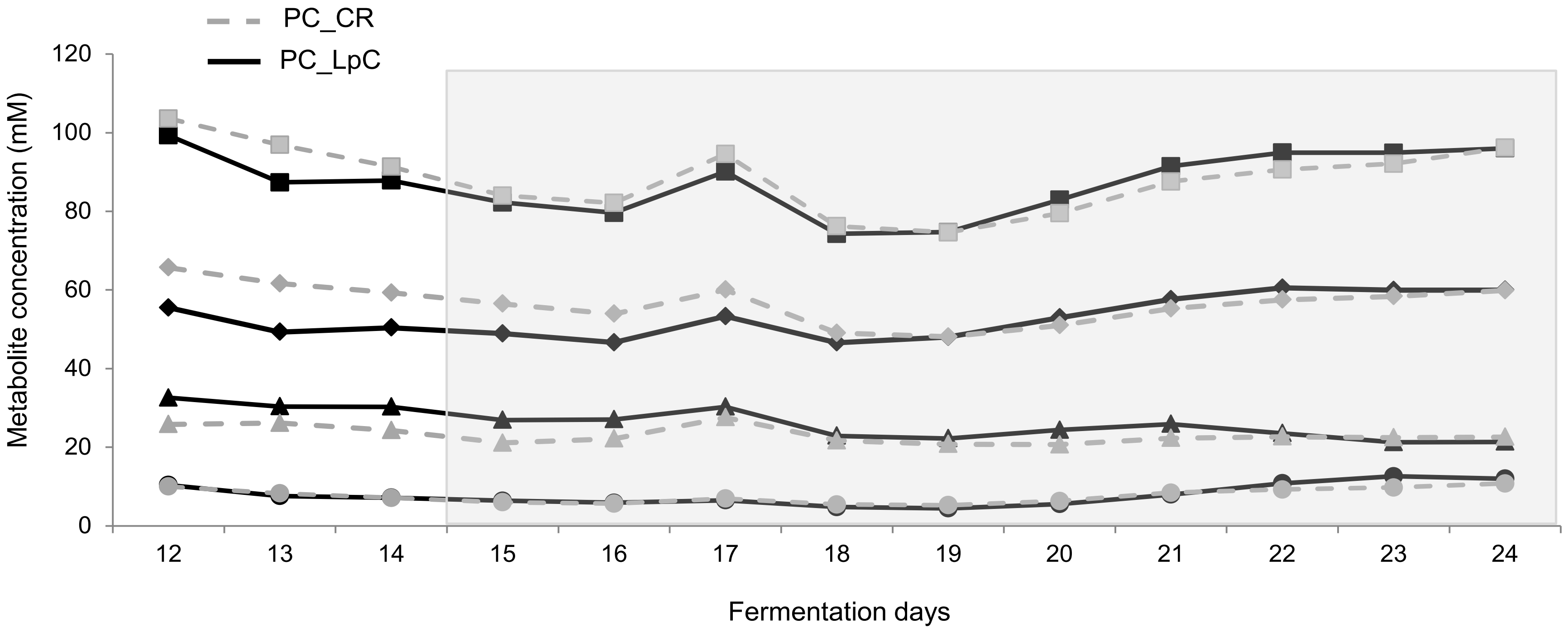


**Figure S 2** Metabolite concentrations in PC_LpC and PC_CR of colonic model 1 assessed with HPLC-RI. *L. paracasei* was added twice daily to PC_LpC starting from day 15. Total metabolites (squares), acetate (diamonds), butyrate (triangles) and propionate (circles).
